# Functional clinical impairments and frailty in interstitial lung disease patients

**DOI:** 10.1183/23120541.00144-2022

**Published:** 2022-10-17

**Authors:** Pierre-François Tremblay Labrecque, Geneviève Dion, Didier Saey

**Affiliations:** 1 Centre de Recherche, Institut Universitaire de Cardiologie et de Pneumologie de Québec, Université Laval, Québec, QC, Canada; 2 These authors contributed equally

## Abstract

**Background:**

Patients with interstitial lung disease (ILD) often present with persistent dyspnoea and reduced exercise capacity and quality of life (QoL), but their functional limitation in relation to their frailty status remains unclear. We thus aimed to compare exercise tolerance, functional mobility, and muscle function and composition between ILD participants and healthy subjects and according to their frailty status.

**Methods:**

A total of 36 ILD participants and 15 heathy subjects performed a 6-min walk test, a 1-min sit-to-stand test, a Short Physical Performance Battery test, a hand grip test and complete quadriceps function testing. Patient-related impacts were assessed *via* questionnaires. Muscle composition was obtained using noncontrast computed tomography scans. The frailty status of patients with ILD was determined using the Fried frailty phenotype assessment.

**Results:**

Compared with control subjects, ILD participants exhibited significantly lower performance in every exercise and functional capacity test, higher dyspnoea and depression scores, and worse QoL. In ILD participants, the same observations were noted for the frail subgroup compared with the robust subgroup. No differences in muscle function and composition were observed between the ILD and control group, but mid-thigh muscle cross-sectional area and skeletal muscle index were significantly reduced in frail ILD participants.

**Conclusions:**

ILD patients present reduced exercise tolerance and functional capacity, and have decreased health-related QoL, when compared with healthy subjects. Physical frailty seems to be associated with worse clinical status, exercise tolerance, muscle and functional impairment, and decreased QoL. The 1-min sit-to-stand test may be a good discriminatory test for frailty status in ILD patients.

## Abstract 2

**Interstitial lung disease patients present clinical, exercise and functional capacity impairments. Patients with worse physical frailty status also present worse exercise tolerance, muscle and functional impairment, and decreased quality of life.** https://bit.ly/3OARUKq

## Introduction

Interstitial lung diseases (ILDs) constitute more than 200 diseases that cause progressive fibrosing of the pulmonary interstitium. The excessive deposition of fibrosing tissue results in reduction of the elastic properties of the lung, reduction of lung volumes, thickening of the alveolar–capillary membrane and gas exchange abnormalities [1, 2]. Consequently, patients with ILD present progressive dyspnoea, diminished exercise tolerance [3] and physical activity levels [4, 5] that reduce significantly their health-related quality of life (HRQoL) [6].

Although evidence on a causal relationship is still missing, several well-established muscle dysfunction promoting factors such as chronic hypoxaemia, inflammatory and oxidative stress, corticosteroid use, physical inactivity, and malnutrition are also frequently reported in ILD patients, and some evidence showed that lower limb muscle atrophy and dysfunction are present in patients with ILD [7].

Multiple tests were designed to characterise exercise tolerance and functional capacity of ILD patients. The 6-min walk test (6MWT) is currently the gold standard for the evaluation of exercise tolerance due to its mortality prediction [3], but other tests such as the 1-min sit-to-stand (1STS) test and Short Physical Performance Battery (SPPB) have been studied with increasing interest in recent years [8–10].

Frailty is described as the vulnerability to adverse outcomes resulting from cumulative declines across multiple systems [11]. The accumulation of functional and perceptual limitations reported in patients with ILD are the main factors in the development of frailty in this population. Frailty predicts mortality in multiple chronic diseases [12, 13] and is associated with an augmentation of healthcare utilisation in COPD [14]. In patients with ILD, frailty has been associated with worse HRQoL and increased hospitalisation and mortality [15]. The prevalence of frailty of 50% recently observed by Milne *et al.* [16] suggests that frailty is common in ILD patients, but little information is available to quantify the nature and severity of functional and exercise limitations, muscle function and composition alterations, perceptual limitations, and QoL of patients with regard to their frailty status. From a rehabilitation perspective, it is clinically relevant to obtain a holistic picture of the ILD patient's functional status. It allows early intervention in implementing home resources and referral to pulmonary rehabilitation programmes that are adapted to the patient's needs [17].

The main objective of the present study was to compare exercise capacity, functional mobility, muscle function and composition, and HRQoL of a group of ILD participants with a group of healthy subjects matched for comparable age and sex. Using the same variables, the secondary objective was to determine if significant differences exist between subgroups of ILD patients when separated according to their frailty status.

## Methods

### Study design and participants

36 consecutive patients with a diagnosis of fibrosing ILD were prospectively and randomly recruited from the outpatient ILD referral centre at the Institut Universitaire de Cardiologie et de Pneumologie de Québec, Université Laval (Québec, QC, Canada) from May 2018 to March 2021. The ILD participants were classified according to frailty status and matched with 15 healthy controls of similar age and sex recruited via our research centre data bank consisting of healthy participants who agreed to be contacted for future studies. Participants of both groups were excluded if they had a history of syncope, significant cardiac disease, or incapacitating musculoskeletal, neurological or rheumatological conditions. ILD participants with any other significant respiratory disease (*i.e.* COPD), a diagnosis of sarcoidosis, a hospitalisation for acute exacerbation of ILD within the last 3 months and having participated in a pulmonary rehabilitation programme in the past 6 months were also excluded. The study was approved by the local ethics committee board (2018–3010, 21595) and all participants signed a consent form before the initiation of study procedures.

### Procedures

The protocol consisted of two visits. At the first visit, age, sex, ILD diagnosis (according to the American Thoracic Society (ATS)/European Respiratory Society (ERS) classification [18]), age at diagnosis, medication and oxygen supplementation were collected from the medical records. Anthropometric data were then collected using a stadiometer for height and weight from which body mass index (BMI) was calculated. Pulmonary function tests, including spirometric testing, plethysmography and measurement of diffusing capacity of the lung for carbon monoxide (*D*_LCO_), were performed conducted in accordance with ATS/ERS guidelines [19] for the ILD subjects, and spirometry was conducted for the participants of the control group. The ILD-GAP (Gender–Age–Physiology) score was calculated from the collected data according to Ryerson *et al.* [20]. After familiarisation with the procedures, participants performed a 1STS test according to the protocol described by Ozalevli *et al.* [21] and a SPPB test according to the National Institute on Aging protocol [22]. The SPPB test consists of the sum of three separate functional components: 1) fastest time to complete 5 times sit-to-stand (5TSTS), 2) 10-s static standing balance test and 3) 4-m walk test [23]. Each component is scored out of 4 for a total of 12 points ranging from 0 (functional impairment) to 12 (maximal functional mobility) [24]. Functional limitation was defined at a cut-off of ≤9 such as described in a previous study [25].

On the second visit, participants completed the remaining tests: a 6MWT (two trials) according to the official ATS/ERS technical standard for field walking tests in chronic respiratory diseases [26], a hand grip test using the Jamar hydraulic hand dynamometer (J.A. Preston Corporation, Clifton, NJ, USA) protocol [27] and a battery of quadriceps muscle function tests with a computerised dynamometer (Biodex System 4; Biodex Medical Systems, Shirley, NY, USA) using test procedures that have been described in detail elsewhere [28]. A rest period of a minimum of 15 min was provided between each test to allow for both cardiorespiratory parameters and dyspnoea perception to return to baseline values. A mid-thigh computed tomography scan was performed at the beginning of the second visit.

Three questionnaires (St George's Respiratory Questionnaire (SGRQ), University of California San Diego Shortness of Breath (UCSD) questionnaire and Center for Epidemiologic Studies-Depression (CES-D) scale) [29] were administered randomly throughout the course of the two sessions. The CES-D questionnaire was completed in its entire form both as part of the HRQoL assessment and the frailty status determination.

Physical frailty was defined using the Fried phenotype model [11, 30], including five criteria: unintentional weight loss, exhaustion, low level of physical activity, slow walking speed and weakness [11]. Participants who fulfilled none of the criteria were considered robust, participants who fulfilled one or two criteria were classified as pre-frail and participants who fulfilled three or more criteria were classified as frail.

A detailed description of all the procedures is available in the supplementary material.

### Statistical analysis and sample size determination

Based on the study of Corrêa *et al.* [31], who described the differences in multiple functional exercise tests between COPD and a control group, we calculated that 15 participants in the control and the pooled ILD group with a power of 80% with α<0.05 would be sufficient.

Continuous variables were analysed using one-way ANOVA. A mixed statistical model following the means procedure permitted us to compare both the ILD *versus* control groups and the frailty divided subgroups using the same model.

Variables were expressed as mean with standard deviation and results were considered significant at p<0.05. Statistical analyses were performed using SAS version 9.4 (SAS Institute, Cary, NC, USA).

## Results

**FIGURE 1:**
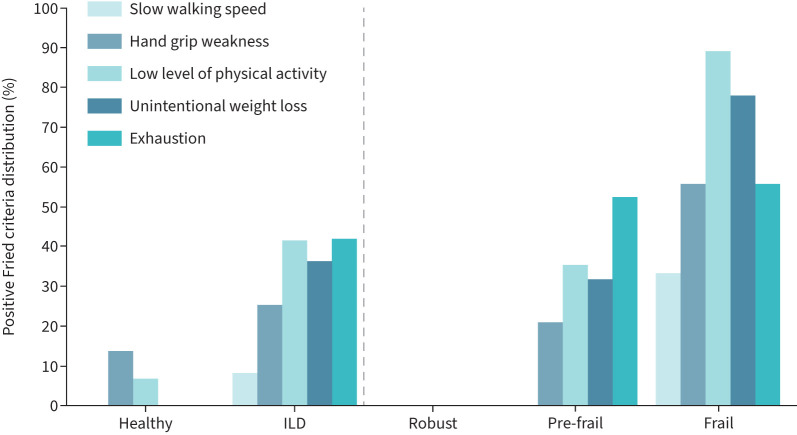
Frailty criteria distribution of the Fried frailty phenotype model in the two different groups (healthy *versus* interstitial lung disease (ILD)). The ILD participants are subdivided into three subgroups according to the number of positive criteria (0: robust; 1–2: pre-frail; ≥3: frail).

Characteristics of ILD participants, controls and the three ILD subgroups in relation to their frailty status are provided in table 1 alongside the diagnosis distribution of the different ILDs. Among participants with ILD, 22% were robust, 53% were pre-frail and 25% were frail. Control and ILD groups were similar in age, sex distribution and BMI. No significant difference was observed for *D*_LCO_ and forced vital capacity (FVC) throughout the frailty phenotypes, although the sample size remain modest for any further interpretation of these parameters. Weekly energy expenditure was significantly lower for frail participants compared with the robust and pre-frail groups. The distribution of the Fried frailty phenotype criteria for each group/subgroup is shown in figure 1. Among the frail criteria, reduced physical activity (89%) and unintentional weight loss (78%) were the most common markers of frailty, followed by exhaustion (CES-D) (56%), hand grip weakness (56%) and slow gait speed (33%).

**TABLE 1:** Characteristics of participants

	**Controls (n=15)**	**ILD (n=36)**	**p-value**	**ILD subgroups**		
				**Robust (n=8)**	**Pre-frail (n=19)**	**Frail (n=9)**
**Age (years)**	69±7	70±7	0.69	67±7^a^	69±8^a^	74±4^a^
**Male (%)**	67	78	0.77	88	74	78
**BMI (kg·m^−2^)**	26±4	28±5	0.09	30±5^a^	29±5^a^	27±7^a^
**Smoking history (pack-years)**	13±15	24±24	0.04	11±10^a^	25±28^a,b^	35±20^b^
**Pulmonary function**						
FVC (L)	3.9±0.5	2.5±0.5	<0.0001	2.8±0.6^a^	2.5±0.4^a^	2.3±0.3^a^
FVC (% pred)	119±17	70±14	<0.0001	77±15^a^	67±10^a^	71±18^a^
*D*_LCO_ (% pred)		51±20		61±11^a^	48±18^a^	49±29^a^
**LTPA-Q (kcal·week^−1^**)	1988±849	877±1044	0.002	1651±1481^a^	877±838^a^	188±192^b^
**Diagnosis distribution**						
IIP						
IPF		17 (47)		3	10	4
NSIP		2 (6)			2	
Unclassifiable		8 (22)		2	3	3
CTD-ILD^#^		7 (19)		3	3	1
HP		1 (3)			1	
Occupational exposure		1 (3)				1
**Time after diagnosis (months)**		49±38		60±50^a^	41±28^a^	53±46^a^
**ILD-GAP score**		3.3±1.8		2.1±1.6^a^	3.3±1.6^a,b^	4.3±1.9^b^
**Medication**						
Steroids		6		1	4	1
Anti-fibrotic agents		13		1	7	5
Immunosuppressants		7		1	5	1
**Oxygen supplementation**		1		0	0	1

Data are presented as mean±sd, n or n (%), unless otherwise stated. ILD: interstitial lung disease; BMI: body mass index; FVC: forced vital capacity; *D*_LCO_: diffusing capacity of the lung for carbon monoxide; LTPA-Q: Leisure Time Physical Activity Questionnaire; IIP: interstitial idiopathic pneumonia; IPF: idiopathic pulmonary fibrosis; NSIP: nonspecific interstitial pneumonia; CTD: connective tissue disease; HP: hypersensitivity pneumonia; GAP: Gender–Age–Physiology. ^#^: CTD-ILD included occupational exposure to asbestos (n=1), rheumatoid arthritis (n=1), scleroderma (n=2) and undifferentiated (n=3). Different superscript letters indicate a statistically significant difference (p<0.05) between subgroups of patients with ILD such that groups with the same superscript letter are statistically similar but different from groups with a different superscript letter.

### Exercise tolerance and functional mobility

The comparison for exercise tolerance and functional mobility between the different subgroups is presented in table 2. The control group performed significantly better than the ILD group for each of the 6MWT, 1STS test and SPPB (including both the 5TSTS and walking speed categories as part of a separate analysis). The 6MWT elicited a slightly more profound desaturation compared with the 1STS test (6MWT 85±4% *versus* 1STS 89±2%; p=0.0003) in the pooled group of ILD participants. Compared with robust participants, frail ILD participants walked a shorter distance on the 6MWT, had a lower number of repetitions on the 1STS test, walked slower on the 4-m walk test and took more time to perform five rises from a chair in the 5TSTS test. The 1STS test was the only test for which a significant difference was observed between each of the subgroups of ILD participants according to their frailty status.

**TABLE 2:** Exercise tolerance and functional capacity

	**Controls (n=15)**	**ILD (n=36)**	**p-value**	**ILD subgroups**		
				**Robust (n=8)**	**Pre-frail (n=19)**	**Frail (n=9)**
**6MWT**						
Distance (m)	615±83	476±94	<0.0001	570±89^a^	477±73^a^	389±50^b^
Distance (% pred)	124±15	101±18	<0.0001	118±11^a^	100±15^b^	87±16^b^
End-test saturation (%)	95±4	85±6	<0.0001	86±6^a^	85±6^a^	85±9^a^
**1STS test**						
Repetitions (n)	30±7	21±4	<0.0001	26±2^a^	21±3^b^	17±3^c^
End-test saturation (%)	96±2	89±5	<0.0001	89±5^a^	90±5^a^	88±4^a^
**5TSTS test time** **(s)**	9.9±2.4	12.9±3.3	0.0019	10.3±1.6^a^	13.0±2.6^b^	15.0±4.3^b^
**SPPB total score**	11.6±0.7	10.5±1.2	0.0007	11.4±0.7^a^	10.5±0.9^a^	9.7±1.6^a^

Data are presented as mean±sd, unless otherwise stated. ILD: interstitial lung disease; 6MWT: 6-min walk test; 1STS: 1-min sit-to-stand; 5TSTS: 5 times sit-to-stand; SPPB: Short Physical Performance Battery. Different superscript letters indicate a statistically significant difference (p<0.05) between subgroups of patients with ILD such that groups with the same superscript letter are statistically similar but different from groups with a different superscript letter.

### Muscle function and composition

The muscle function and composition data (table 3) showed no difference in hand grip strength measurements between the control and ILD groups, but also within the three frailty subgroups of ILD participants. No difference was observed in quadriceps measurements when the pooled ILD group was compared with the control group, but all quadriceps functions (power, strength and endurance) were significantly lower in the frail subgroup when compared with both the pre-frail and robust subgroups.

**TABLE 3:** Muscle function and composition

	**Controls (n=15)**	**ILD (n=36)**	**p-value**	**ILD subgroups**		
				**Robust (n=8)**	**Pre-frail (n=19)**	**Frail (n=9)**
**Hand grip (kg)**	37±12	34±8	0.37	38±7^a^	34±9^a^	31±8^a^
**Quadriceps**						
Peak torque (Nm)	107±26	95±27	0.10	111±26^a^	100±22^a^	70±24^b^
MVC (Nm)	148±45	141±38	0.57	170±37^a^	144±32^a^	106±21^b^
Endurance (J)	2424±743	2212±620	0.27	2631±603^a^	2290±502^a^	1620±469^b^
**CT scan**						
Mid-thigh muscle CSA (cm^2^)	245±42	231±50	0.33	271±43^a^	232±46^a,b^	194±35^b^
Skeletal muscle index (cm^2^·m^−2^)	85±12	79±23	0.37	96±15^a^	77±26^a,b^	70±15^b^
Attenuation (mean HU)	44±4	41±5	0.01	42±5^a^	42±5^a^	39±5^a^

Data are presented as mean±sd, unless otherwise stated. ILD: interstitial lung disease; MVC: maximal voluntary contraction; CT: computed tomography; CSA: cross-sectional area. Different superscript letters indicate a statistically significant difference (p<0.05) between subgroups of patients with ILD such that groups with the same superscript letter are statistically similar but different from groups with a different superscript letter.

While presenting similar subcutaneous adipose tissue and total muscle cross-sectional area (CSA), ILD participants exhibited significantly reduced muscle attenuation and higher deep adipose tissue of the mid-thigh CSA when compared with the control group.

### Dyspnoea, depression and HRQoL questionnaires

The results of the UCSD, CES-D and SGRQ questionnaires for the ILD and control groups are presented in table 4. The three questionnaires exhibited, respectively, significantly higher reported dyspnoea, higher score of depression and higher score on SGRQ representing worse HRQoL for the ILD group. The same observations were seen for frail participants compared with robust participants.

**TABLE 4:** Dyspnoea, depression and health-related quality of life questionnaires

	**Controls (n=15)**	**ILD (n=36)**	**p-value**	**ILD subgroups**		
				**Robust (n=8)**	**Pre-frail (n=19)**	**Frail (n=9)**
**UCSD score**	8±8	42±24	<0.0001	16±7^a^	46±23^b^	56±17^b^
**CES-D score**	3±5	10±9	0.003	4±5^a^	12±10^b^	12±7^a,b^
**SGRQ score**						
Total	4±7	46±18	<0.0001	32±14^a^	47±18^a^	57±14^b^
Symptoms	12±20	56±24	<0.0001	47±16^a^	54±27^a^	69±21^a^
Activities	7±10	75±22	<0.0001	49±19^a^	80±17^b^	88±14^b^
Impact	2±6	48±26	<0.0001	32±19^a^	47±27^a,b^	62±21^b^

Data are presented as mean±sd, unless otherwise stated. ILD: interstitial lung disease; UCSD: University of California San Diego Shortness of Breath Questionnaire; CES-D: Center for Epidemiologic Studies-Depression scale; SGRQ: St George's Respiratory Questionnaire. Different superscript letters indicate a statistically significant difference (p<0.05) between subgroups of patients with ILD such that groups with the same superscript letter are statistically similar but different from groups with a different superscript letter.

## Discussion

This study shows that ILD patients present significantly reduced exercise tolerance and functional capacity, higher dyspnoea, and reduced HRQoL compared with control subjects. Although ILD participants present some muscle composition alterations (low attenuation), muscle function seems preserved. When the cohort of ILD participants is subdivided into their respective frailty status phenotypes, the exercise and functional test performance as well symptoms of dyspnoea, depression and QoL significantly worsen as frailty status progresses, which may emphasise cumulative declines across multiple systems.

### Exercise tolerance and functional mobility

ILD participants included in the study had lower weekly physical activity and presented worse performance on all of the exercise tolerance and functional tests compared with the control group. Although our ILD participants presented preserved 6MWD (476±94 m), this performance was significantly worse compared with control healthy subjects (615±83 m). The walk distance for our ILD participants is consistent with the upper limit of previously published data ranging from 377 to 487 m [32–35] and is also similar to the mean walk distance in major therapeutic studies in idiopathic pulmonary fibrosis (IPF) (397–420 m). The mean number of 1STS repetitions (21±4 repetitions) for our ILD group was significantly lower than the control healthy subjects (30±7 repetitions) and was similar to other studies in ILD participants (21–22 repetitions) [8, 9]. The slightly more profound desaturation at the end of the 6MWT when compared with the 1STS test is also consistent with previously reported results in ILD populations [8]. This confirms the poor exercise tolerance and functional capacity of the ILD participants compared with the healthy subjects.

When the ILD participants were classified according to their frailty status, performance for the 6MWT, 1STS test and 5TSTS test was significantly lower between the frail and robust subgroups in ILD participants, but no difference was observed between subgroups of patients for the total score of the SPPB, for which the performance ceiling is probably too low to adequately assess the ILD population. The 1STS test results allowed the best differentiation between the frail subgroups since each of the frailty-separated subgroups was significantly different from each other. Nonetheless, even if the 6MWT did not allow this differentiation, it effectively detected the frail participants from the robust participants. No significant differences were observed for FVC, *D*_LCO_ and the level of end-test saturation on functional and exercise tests between the frailty-separated ILD subgroups. Although these tests might be good indicators of the presence and progression of ILDs [36, 37], these clinical markers may be poor follow-up indicators for the detection of frailty in the ILD population.

### Muscle function and composition

Results concerning hand grip tests remain conflicting in the literature. Even if some previous studies [38, 39] did not find significant weakness in hand grip testing (95–97% predicted value) in patients with ILD in accordance with our results, at least two other studies presented reduced muscle function in ILD patients [40, 41]. Although hand grip was part of the frailty assessment, the mild differences between the results of the frailty subgroups did not reach statistical significance. This suggests that hand grip might not be an effective tool to discriminate frailty phenotypes in ILD patients. Additional data will be necessary before making any conclusions about this subject.

Lower limb function seems preserved in robust and pre-frail patients, and only appears to be affected in frail ILD patients. Some ILDs, such as IPF, progress rapidly and have a worse prognosis. Since physical inactivity and secondary muscle composition alterations usually develop over time, this may contribute to explain the absence of significant differences in muscle function between the ILD and control groups.

Contrary to the extensive study of Maddocks *et al*. [42], who reported lower muscle CSA in COPD subjects while density and intramuscular fat showed insignificant differences, our ILD participants exhibit similar macroscopic muscle CSA compared with the control group, but increased intramuscular fat infiltration (as portrayed by the lower attenuation, which reflects the lower muscle fibre quality). This correlates with the Guler *et al.* [43] study that showed a higher fat mass in ILD individuals with more impaired pulmonary function. These muscle composition alterations are particularly interesting considering there is a considerable overlap between frailty and muscle alterations [44] that our data seem to confirm in ILD frail patients. The loss of muscle function and mass as well as alteration of muscle composition occur with ageing [45] and in other respiratory chronic disease, such as COPD [46]. Mechanisms underlying alterations of lower limb muscle function and composition are complex and interrelated, as addressed in a recent joint ERS/ATS statement in patients with chronic respiratory diseases [46]. Despite the fragmentary data from a very heterogeneous groups of patients and respectful of the fact that this study was not designed to address this question, muscle alterations in some patients could be a consequence of systemic disease such as connective tissue or inflammatory disease [47]. In addition, muscle alterations in frail ILD patients may result from advanced age and the consequences of disease, such as chronic hypoxaemia, inflammatory and oxidative stress, corticosteroid use, physical inactivity, and malnutrition that may exert a synergistic, deleterious effect on muscle function [7].

It is also interesting to observe that impairment of several markers of functional capacity (6MWT (% predicted distance), 1STS test (repetitions) and 5TSTS test (time)) and patient-related impacts are present in pre-frail ILD patients. Although larger and adequately designed studies are needed to confirm this hypothesis, our data suggest that the alterations of functional physical performance precede muscle alterations.

### Dyspnoea, depression and HRQoL questionnaires

As expected, ILD participants had a significantly higher level of dyspnoea. Our results also showed a significantly higher prevalence of depression symptoms in ILD participants than the control group. If the cut-off of ≥16 points was used to identify individuals at risk of clinical depression on the CES-D questionnaire, 28% of the ILD participants were identified as at risk of clinical depression [38]. This is much higher than the reported depression prevalence in the general population (3.8–12.6%), which highlights the multidisciplinary needs in evaluating ILD diseases [48].

Finally, ILD participants had higher scores on the SGRQ compared with control healthy subjects. This result confirms that HRQoL is profoundly impaired in patients with ILD. Scores on the SGRQ were comparable to previous studies in IPF and a comparable population [49].

### Methodological considerations and study limitation

This study raises some methodological considerations and limitations. The first concerns the sample size that was determined in order to compare ILD patients with healthy subjects. Interpretation between the subgroups of ILD patients according to their frailty status must be made with caution due to the limited number of patients in each group, and should be validated by a larger and adequately powered study.

Regarding the recruitment of participants, the consecutive and randomly selected patient recruitment strategy and classification according to their frailty status used in the study ensures having a representative group of ILD patients. It is thus reassuring that the group of patients is similar in terms of previously published data, both for their clinical and respiratory status and for their frailty status distribution [50].

In addition, since the control participants were recruited *via* our research centre data bank, we are aware of a possible social participation bias explaining the high performance of our control group for respiratory function and functional exercise capacity. However, with regard with the huge magnitude of the between-group (ILD *versus* healthy subjects) values, the authors are confident that the observed differences were only slightly altered by this potential bias and that the impairments described in the present study are indeed present in ILD patients when compared with healthy subjects of comparable age, sex and BMI.

### Clinical implications

Aside from significant muscle dysfunction, the ILD population was less active and presented decreased exercise tolerance and functional status, a higher level of dyspnoea, fatigue, anxiety and depressive symptoms, and a lower QoL compared with the control population, especially in patients with frail status. This is clinically relevant because all of these concerns may be addressed by pulmonary rehabilitation, which includes exercise training and behaviour change interventions [51]. The most recent review on pulmonary rehabilitation outcomes [52] puts emphasis on the potential benefits in improving functional performance and QoL (moderate certainty) and maximum exercise capacity, dyspnoea and long-term survival (low certainty) in ILD patients. In COPD, frail patients respond favourably to pulmonary rehabilitation [17] and a similar effect could be expected in a population with ILD, although data are still missing for this population [53]. Because of the high reported prevalence of frailty in patients with ILD [50, 53], it is thus crucial to identify and understand the multidimensional concerns of these patients in order to individualise the intervention to suit their needs and priorities [54].

Finally, from the novel perspective brought by our findings, the 1STS test appears to be a good discrimination test to assess frailty status in the ILD population. In this regard, the cut-off for the number of repetitions for which the 1STS is predictive of frail status would be an interesting topic for further studies.

### Conclusions

Despite relatively preserved muscle function and structure, ILD patients are less active, present reduced exercise tolerance and functional capacity, a higher level of dyspnoea, fatigue, anxiety and depressive symptoms, and have decreased HRQoL when compared with healthy subjects of comparable age and sex distribution. In patients with ILD, physical frailty seems associated with worse clinical status, exercise tolerance, muscle and functional impairment, and decreased QoL and leisure time physical activity. The 1STS test may be a good discriminatory test for frailty status in ILD patients.

## Supplementary material

10.1183/23120541.00144-2022.Supp1 **Please note:** supplementary material is not edited by the Editorial Office, and is uploaded as it has been supplied by the author.
Supplementary material 00144-2022.supplement
