# Supplementary material for: Functional clinical impairments and frailty in interstitial lung disease patients
Source: ERJ Open Res. 2022 Oct 17;8(4):00144-2022. doi: 10.1183/23120541.00144-2022 (PMC9574552; doi:10.1183/23120541.00144-2022)
Supplement: Supplementary file 1 [file 00144-2022.supplement.pdf]

Functional Clinical Impairments and Frailty in Interstitial Lung Diseases Patients

Pierre-François Tremblay Labrecque[1*], Geneviève Dion[1*] and Didier Saey[1]

[1]Centre de Recherche, Institut universitaire de cardiologie et de pneumologie de Québec, Université Laval, Québec, Canada.

* *These authors contributed equally to the manuscript*

ONLINE SUPPLEMENTARY MATERIAL 1

DETAILED PROCEDURES

Study design and participants

36 consecutive patients with a diagnosis of fibrosing ILD were recruited from an outpatient ILD referral center at the *Institut universitaire de cardiologie et de pneumologie de Québec*, Université Laval (Québec, Canada) from May 2018 to March 2021. Participants of the ILD group were recruited according to their frailty status, to represent the frailty distribution of the clinic (data awaiting publication). The ILD participants were matched with 15 healthy controls of similar age and sex. Participants of both groups were excluded if they had a history of syncope, significant cardiac disease or incapacitating musculoskeletal, neurological, or rheumatological conditions. ILD participants with any other significant respiratory disease (i.e. chronic obstructive pulmonary diseases (COPD)), a diagnosis of sarcoidosis, a hospitalization for acute exacerbation of ILD within the last 3 months, and having participated in a pulmonary rehabilitation program in the past 6 months were also excluded. The study was approved by the local ethic committee board (N 2018-

3010, 21595) and all participants signed a consent form before the initiation of study procedures.

Procedures

The protocol consisted of two visits. At the first visit, age, sex, ILD diagnosis (according to the ATS/ERS classification) and age at diagnosis were collected from the medical records. Anthropometric data was then collected using a stadiometer for the height weight from which the BMI was calculated. Pulmonary function tests were also performed and after a familiarization to the procedures, participants executed the Short Physical Performance Battery (SPPB) and the 1STS. On the second visit, participants completed the remaining tests: 6MWT (2 trials), the hand grip and quadriceps muscle function tests. A rest period of a minimum of 15 minutes was provided between each test to allow for both the cardiorespiratory parameters and dyspnea perception to return to baseline values. Questionnaires were administered randomly throughout the course of the two sessions and a mid-thigh computed tomodensitometry (CT scan) of the thigh was performed at the beginning of the second visit.

Pulmonary function

Pulmonary function including spirometric testing, plethysmography and measurement of the diffusing capacity for carbon monoxide (DL_{CO}) was conducted in accordance with the ATS/ERS guidelines [1] for the ILD subjects. Data were reported as % of predicted values using ERS Global Lung Function Initiative reference equations. [2] For participants who had realized those tests as part of their required medical follow-up in the last 3 months, the pulmonary function tests were not repeated and the result of their recent exam were used instead. Participants of the control group performed only the spirometry to rule-out any abnormalities suggestive of lung disease.

Physical Frailty

Physical frailty was defined using the Fried phenotype model, [3] [4] including five criteria: unintentional weight loss, exhaustion, low level of physical activity, slow walking speed and weakness. The unintentional body mass loss history ≥4.5 kg was assessed by answering "yes" to the question: "In the last year, have you lost more than 10 pounds unintentionally (i.e., not due to dieting or exercise)?" Self-reported exhaustion was assessed by asking to participants two questions of the Center for Epidemiologic Studies Depression Scale (CES-D) [39] : In the las week: (a) I felt that everything I did was an effort; (b) I could not get going. Criteria for exhaustion was met if the participant answer that he felt one of this way for 3 or more days in the last week. Low physical activity criteria was reached if the reported physical activity level was <383 Kcals for men or <270 Kcals for women base on the short version of the Minnesota Leisure-Time Physical Activity Questionnaire (LTPA-Q). The LTPA-Q consisted of a list of 26 activities to which the patient recalled its participation during the past year. Each activity was associated with an intensity score. The total score (Kcal/week of expenditure) was used to determine the frailty positive criteria score related to physical activity. [3] Slow walking speed and weakness were assessed by the 4-meter gait speed test (4MGS; slow walk) and handgrip dynamometry (weakness), respectively and positive criteria were determined via previously published cutoffs stratified by gender and height. [3] Participants who fulfilled none of the criteria were considered robust, participants who fulfilled 1 or 2 criteria were classified as pre-frail, and participants who fulfilled ≥3 criteria were classified as frail.

Exercise tolerance

Exercise tolerance was assessed with the 6MWT and the 1STS tests.

6-minute walk test (6MWT). The 6MWT was performed according to the official ATS/ERS technical standard field walking tests in chronic respiratory diseases.[5] The prediction values were used from the equations of Enright et al. [6] The test was performed indoor, along a flat, straight, 30-meter long corridor with two cones placed 0.5 meters from the extremities to mark the turning points in the course. Participants were systematically instructed to walk the longest distance possible back and forth, around the cones in 6 minutes. The best result of two separate trials was kept for analysis.

1-minute sit-to-stand. The 1STS tests were completed according to the protocol described by Ozalevli et al.[7] and supervised by trained research staff. A standardized 48 cm chair without armrest was used and positioned against a wall. The participant was positioned with their knees at a 90° angle, both feet on the floor and arms crossed around the chest and was instructed to stand-up completely and to sit back down as many times possible within one minute, without using their hands. The pace of the test was determined by the participant. It was not mandatory to fully sit back on the chair, but the back had to reach vertical. Participants were informed of the time when 15 seconds were left but no encouragement was provided during the test. The number of fully completed repetitions within the minute was used for analysis.

Functional mobility

Short performance physical battery test (SPPB). Functional mobility was assessed with the SPPB performed according to the National Institute on Aging protocol. [8] The SPPB test consist of the sum of three separate functional components. 1. Fastest time to complete 5 times sit-to-stand

(5TSTS): rising from a chair with their arms across their chest for 5 repetitions. The test environment is the same as the one previously described in the 1STS section. 2. 10-sec static standing balance tests requiring participants to maintain each of three stances for 10 seconds with feet in three positions (feet placed side-by-side, semi-tandem, and in tandem). 3. 4-meter walk test performed at usual speed. [9] Each component is scored on 4 for a total of 12 points ranging from 0 (functional impairment) to 12 (maximal functional mobility).[10] Functional limitation was defined at a cutoff of ≤ 9 such as described in a previous study. [11]

Muscle composition and function

Muscle function was tested for both the upper and lower limbs via the hand grip strength and quadriceps muscle testing, respectively. The muscle composition was obtained using the computed tomography scans.

Hand grip strength. Hand grip strength was tested using The Jamar® hydraulic hand dynamometer (J. A. Preston Corporation,Clifton, NJ) protocol [12] which requires the participant to be seated, elbow flexed at 90º, wrist between 0º and 30º and between 0º and 15º of ulnar deviation. The participant was then instructed and verbally reinforced to squeeze the dynamometer at his maximal strength. 3 trials separated of 15 seconds were performed for each hand. The best of two reproducible trials was used for analysis.

Quadriceps muscle function. Quadriceps strength, power and endurance were measured with a computerised dynamometer (Biodex System 4 Biodex Medical Systems, Shirley, New York, NY, USA) using test procedures that have been described in detail elsewhere [13].

Quadriceps strength was measured during a maximal voluntary contraction (MVC) using an isometric (static) protocol at a 90° knee angle [13], performed in line with international

recommendations [14]and reported in Newton-meters (Nm).

Quadriceps power, and endurance were obtained during a 30 maximal isokinetic contractions protocol of 30 maximal knee extensions throughout the full range of movement at 90° per second, with passive knee flexion in which the total work (J) and peak torque (Nm) were considered such as endurance and power respectively.

Muscle composition.

The mid-thigh muscle surface and attenuation data was obtained using non-contrast computed tomography scans (CT scan) at the mid femur level. The scan parameters were a voltage of 140 kilovolts (kV) measured according to the weight of the subject, an acquisition load (number of X-rays) fixed to 200 milliampere-seconds (mAs) and a five millimeters slice thickness (mm). All images were assessed using specialized image analysis software (Slice-O-Matic, Tomovision, Montréal, Québec, Canada) and according to standardized techniques. [15] Muscle tissue areas (cm2) were computed using an attenuation range of -29 to 150 HU, such as recommended. [16] Since skeletal muscle with relatively lower attenuation contain proportionally more adipose tissue [17], muscle tissue corresponding to attenuation range of -29 to 34 HU was considerate as low attenuation muscle and muscle tissue between 35 and 150 HU as normal attenuation muscle. Skeletal muscle index (SMI) was obtained after CSA indexation for height (CSA/height^2). [18]

Questionnaires

Three self-administered questionnaires were used to assess the patient-reported impacts associated with ILD. The St-George's Respiratory Questionnaire (SGRQ) contains 76 items separated in three sections: Symptoms (SGRQ-S), Activities (SGRQ-A) and Impacts (SGRQ-I) which are

summarized into a total score. [19] Even though the SGRQ was originally developed for COPD [20], its psychometric properties were also addressed in several IPF studies which concluded to its adequacy in measuring health-related quality of life. [21] [22] The University of California San Diego Shortness of Breath questionnaire (UCSD) contain 21 various activities which the participant rates his dyspnea from 0 (“Not at all”) to 5 (“Maximally or unable to do because of breathlessness”) and 3 items regarding the perceived impact of the breathlessness. [23] Similar studies concluded to its validity to adequately assess dyspnea in both IPF [23] and heterogeneous groups of fibrotic ILD patients [24]. The CES-D scale is a 20-item questionnaire which aims to screen depression symptomatology. [25] This last questionnaire was completed in its entire form both as part of the HRQoL assessment and the frailty status determination process described below.

Statistical analysis and sample size determination

Based on the study of Corrêa et al. [26] who described the differences in multiple functional exercise tests between chronic obstructive pulmonary disease and a control group, we calculated that 15 participants in the control and the pooled ILD group with a power of 80% with an alpha of $\alpha < 0.05$ would be sufficient.

Continuous variables were analyzed using one-way ANOVA. Differences between ILD and control groups and frailty divided subgroups analysis. A mixed statistical model following the means procedure permitted to compare both the ILD vs. control groups and the frailty divided subgroups using the same model. The Satterthwaite’s degree of freedom statement was added for variables analyzed using unequal variance structures. Posteriori comparisons were performed using the Tukey’s technique.

Receiver operating characteristics (ROC) curves were created to analyze the sensitivity, specificity, area under the curve (AUC) and the positive and negative likelihood ratio (LR) of SPPB cutoff

points to detect physical frailty. The odds ratios of various cutoff points of these tests to predict physical frailty were also calculated along with their respective confidence intervals.

Variables were expressed as mean ± standard deviation and results were considered significant at $p < 0.05$. Statistical analyses were performed using the SAS version 9.4 (SAS Institute Inc, Cary, NC).

Bibliography

1. Culver BH, Graham BL, Coates AL, et al. Recommendations for a Standardized Pulmonary Function Report. An Official American Thoracic Society Technical Statement. *Am J Respir Crit Care Med.* 2017;196(11):1463-1472.
2. Quanjer PH, Stanojevic S, Cole TJ, et al. Multi-ethnic reference values for spirometry for the 3-95-yr age range: the global lung function 2012 equations. *Eur Respir J.* 2012;40(6):1324-1343.
3. Fried LP, Tangen CM, Walston J, et al. Frailty in older adults: evidence for a phenotype. *J Gerontol A Biol Sci Med Sci.* 2001;56(3):M146-156.
4. Crocker TF, Brown L, Clegg A, et al. Quality of life is substantially worse for community-dwelling older people living with frailty: systematic review and meta-analysis. *Qual Life Res.* 2019;28(8):2041-2056.
5. Holland AE, Spruit MA, Troosters T, et al. An official European Respiratory Society/American Thoracic Society technical standard: field walking tests in chronic respiratory disease. *Eur Respir J.* 2014;44(6):1428-1446.
6. Enright PL, Sherrill DL. Reference equations for the six-minute walk in healthy adults. *Am J Respir Crit Care Med.* 1998;158(5 Pt 1):1384-1387.
7. Ozalevli S, Ozden A, Itil O, Akkoclu A. Comparison of the Sit-to-Stand Test with 6 min walk test in patients with chronic obstructive pulmonary disease. *Respir Med.* 2007;101(2):286-293.
8. Guralnik JM, Simonsick EM, Ferrucci L, et al. A short physical performance battery assessing lower extremity function: association with self-reported disability and prediction of mortality and nursing home admission. *J Gerontol.* 1994;49(2):M85-94.
9. Kon SS, Patel MS, Canavan JL, et al. Reliability and validity of 4-metre gait speed in COPD. *Eur Respir J.* 2013;42(2):333-340.
10. van den Berg M, Sherrington C, Killington M, et al. Video and computer-based interactive exercises are safe and improve task-specific balance in geriatric and neurological rehabilitation: a randomised trial. *J Physiother.* 2016;62(1):20-28.
11. Patel MS, Mohan D, Andersson YM, et al. Phenotypic characteristics associated with reduced short physical performance battery score in COPD. *Chest.* 2014;145(5):1016-1024.
12. Mathiowetz V. Comparison of Rolyan and Jamar dynamometers for measuring grip strength. *Occup Ther Int.* 2002;9(3):201-209.
13. Frykholm E, Gephine S, Saey D, et al. Inter-day test-retest reliability and feasibility of isokinetic, isometric, and isotonic measurements to assess quadriceps endurance in people with chronic obstructive pulmonary disease: A multicenter study. *Chron Respir Dis.* 2019;16:1479973118816497.
14. Maltais F, Decramer M, Casaburi R, et al. An official American Thoracic

Society/European Respiratory Society statement: update on limb muscle dysfunction in chronic obstructive pulmonary disease. *Am J Respir Crit Care Med.* 2014;189(9):e15-62.

15. Baracos VE, Reiman T, Mourtzakis M, Gioulbasanis I, Antoun S. Body composition in patients with non-small cell lung cancer: a contemporary view of cancer cachexia with the use of computed tomography image analysis. *Am J Clin Nutr.* 2010;91(4):1133s-1137s.

16. Kazemi-Bajestani SM, Mazurak VC, Baracos V. Computed tomography-defined muscle and fat wasting are associated with cancer clinical outcomes. *Semin Cell Dev Biol.* 2016;54:2-10.

17. Goodpaster BH, Carlson CL, Visser M, et al. Attenuation of skeletal muscle and strength in the elderly: The Health ABC Study. *J Appl Physiol (1985).* 2001;90(6):2157-2165.

18. Albano D, Messina C, Vitale J, Sconfienza LM. Imaging of sarcopenia: old evidence and new insights. *Eur Radiol.* 2020;30(4):2199-2208.

19. Jones PW, Quirk FH, Baveystock CM. The St George's Respiratory Questionnaire. *Respir Med.* 1991;85 Suppl B:25-31; discussion 33-27.

20. Jones PW, Quirk FH, Baveystock CM, Littlejohns P. A self-complete measure of health status for chronic airflow limitation. The St. George's Respiratory Questionnaire. *Am Rev Respir Dis.* 1992;145(6):1321-1327.

21. Swigris JJ, Wilson H, Esser D, et al. Psychometric properties of the St George's Respiratory Questionnaire in patients with idiopathic pulmonary fibrosis: insights from the INPULSIS trials. *BMJ Open Respir Res.* 2018;5(1):e000278.

22. Swigris JJ, Esser D, Conoscenti CS, Brown KK. The psychometric properties of the St George's Respiratory Questionnaire (SGRQ) in patients with idiopathic pulmonary fibrosis: a literature review. *Health Qual Life Outcomes.* 2014;12:124.

23. Swigris JJ, Han M, Vij R, et al. The UCSD shortness of breath questionnaire has longitudinal construct validity in idiopathic pulmonary fibrosis. *Respir Med.* 2012;106(10):1447-1455.

24. Chen T, Tsai APY, Hur SA, et al. Validation and minimum important difference of the UCSD Shortness of Breath Questionnaire in fibrotic interstitial lung disease. *Respir Res.* 2021;22(1):202.

25. Lewinsohn PM, Seeley JR, Roberts RE, Allen NB. Center for Epidemiologic Studies Depression Scale (CES-D) as a screening instrument for depression among community-residing older adults. *Psychol Aging.* 1997;12(2):277-287.

26. Corrêa KS, Karloh M, Martins LQ, dos Santos K, Mayer AF. Can the Glittre ADL test differentiate the functional capacity of COPD patients from that of healthy subjects? *Rev Bras Fisioter.* 2011;15(6):467-473.
